# Terahertz Pulse Emission from Semiconductor Heterostructures Caused by Ballistic Photocurrents

**DOI:** 10.3390/s21124067

**Published:** 2021-06-12

**Authors:** Vitaly Leonidovich Malevich, Pavel Aliaksandravich Ziaziulia, Ričardas Norkus, Vaidas Pačebutas, Ignas Nevinskas, Arūnas Krotkus

**Affiliations:** 1 Stepanov Institute of Physics, National Academy of Science, Nezavisimosti Avenue 68, 220072 Minsk, Belarus; v.malevich@ifanbel.bas-net.by; 2 Belarusian State University of Informatics and Radioelectronics, P. Browki Str. 6, 220013 Minsk, Belarus; 3 Belarusian State University, Nezavisimosti Avenue 4, 220030 Minsk, Belarus; zezyulya@bsu.by; 4 Center for Physical Sciences and Technology, Sauletekio av. 3, LT-10257 Vilnius, Lithuania; vaidas.pacebutas@ftmc.lt (V.P.); ignas.nevinskas@ftmc.lt (I.N.); arunas.krotkus@ftmc.lt (A.K.)

**Keywords:** THz, THz emission spectroscopy, heterojunction, GaInAsBi/InP, band offset, THz pulse generation, ballistic electrons, MBE

## Abstract

Terahertz radiation pulses emitted after exciting semiconductor heterostructures by femtosecond optical pulses were used to determine the electron energy band offsets between different constituent materials. It has been shown that when the photon energy is sufficient enough to excite electrons in the narrower bandgap layer with an energy greater than the conduction band offset, the terahertz pulse changes its polarity. Theoretical analysis performed both analytically and by numerical Monte Carlo simulation has shown that the polarity inversion is caused by the electrons that are excited in the narrow bandgap layer with energies sufficient to surmount the band offset with the wide bandgap substrate. This effect is used to evaluate the energy band offsets in GaInAs/InP and GaInAsBi/InP heterostructures.

## 1. Introduction

When photoexcited by femtosecond optical pulses, most materials emit ultrashort pulses of electromagnetic radiation containing frequencies at the terahertz (THz) frequency range. THz pulse emission has been observed from semiconductors [1], dielectric crystals [2], metals [3], and even from gases [4], and liquids [5]. This universal effect became an effective and popular tool for a contact-less investigation of various materials. Moreover, some semiconductor or ferromagnetic material structures that are most efficient surface emitters can replace photoconductive antennas as the THz radiation sources in the time-domain spectroscopy (TDS) systems, especially in those that are activated by long-wavelength femtosecond lasers [6]. As yet, the most efficient THz pulse emitting surfaces are the narrow bandgap group A3B5 semiconductors such as InAs or InSb [7,8]. This is because the photoexcited electrons and holes move away from the illuminated surface at different velocities, which creates fast changing electrical dipole radiating THz waves. During the first few hundreds of femtoseconds, when the dipole is developing, the photoexcited electrons in narrow-gap A3B5 semiconductors are moving ballistically [9] over the distances reaching several hundred nanometers [10]. Due to the electrons not being scattered during this stage, the initial optical orientation of their momenta [11] is conserved and it is also reflected in the azimuthal angle dependences of THz emission for certain photoexcited crystal planes [12,13].

THz emission due to ballistic photoexcited electron propagation has been exploited for the measurements of conduction band offsets in GaAsBi/GaAs [14] and GaInAsBi/InP [15] heterojunctions. Femtosecond optical pulses with a tunable central wavelength were illuminating the interface between the air and the narrower bandgap material layer. The electrons excited at this interface with lower energy photons could not move farther than the thickness of the narrow-gap layer, and the amplitude of a THz pulse was relatively small. The THz pulse amplitude started to increase only when the excess energy of ballistic electrons was higher than the conduction band offset at the heterojunction between the layer and the wider bandgap substrate. Therefore, the heterostructure offset values could be directly found from the THz excitation spectra measurement. In fact, this band offset measurement technique is an optical equivalent of the so-called ballistic-electron-emission-microscopy (BEEM) [16], where subsurface electronic structure is probed by electrons injected into material from differently biased scanning tunneling microscope tip.

From what is said above, it is clear that femtosecond photoexcitation and THz pulse emission can be used to study the ballistic electrons in semiconductors. One of the important objects of such a study could be the realization of optics-like effects in electronic systems. Ballistic electrons are coherent de Broglie waves that can be reflected or refracted at the heterointerfaces between different semiconductors, much like the electromagnetic waves [17,18]. Their behavior would be exposed more unanimously, if the electrons would be generated at the proximity of the interface rather than away from it, at the air/semiconductor boundary. In this contribution, we present the investigation of THz emission from femtosecond laser-excited heterostructures between InP substrate and GaInAs and GaInAsBi epitaxial layers. Several nontrivial characteristics of this effect, such as different THz excitation spectra dependences and different tilt angles of THz electrical dipoles created after illuminating the structure from the substrate and the layer sides were observed. These differences have been explained by the influence of quasi-ballistic photoelectrons that are entering into the substrate and are refracted there at larger angles fostering more efficient THz radiation outcoupling from the semiconductor structure.

## 2. Materials and Methods

The heterostructures were grown by molecular-beam-epitaxy (MBE) on semi-insulating (001) InP:Fe substrates. Two heterostructures’ samples were investigated: a sample with lattice-matched 600 nm thick Ga_0.47_In_0.53_As layer on InP (sample A) and Ga_0.47_In_0.53_As_1−x_Bix/InP structure with 4% Bi (sample B). Native oxides were desorbed by heating InP substrates at 500 °C under arsenic flux and monitoring the process by observation of a distinctive (2 × 4) surface reconstruction pattern by the reflection high energy electron diffraction. The GaInAs layer was grown at standard growth conditions keeping high substrate temperature at T ~490 °C and the beam equivalent pressure (BEP) ratio As_2_/Ga from 7 to 10. The growth temperature was adjusted by a thermocouple-based controller calibrated by the use of melting points of Sn and InSb. In order to enhance the bismuth incorporation into the growing layer and to avoid its segregation on the surface, the growth of the Ga_0.47_In_0.53_As_0.96_Bi_0.04_ layer was performed at substrate temperature of 240 °C, which is significantly lower than typical MBE growth temperatures for GaInAs. The BEP of group 3 and 5 atomic flux ratio was calibrated by monitoring the surface reconstruction change on separate substrate before the samples’ growth. The growth rate of bismide layer was 0.6 μm/h. The atomic composition of the GaInAsBi layer was determined from X-ray diffraction (XRD) measurements, carried out with Smart Lab Rigaku diffractometer (Rigaku corporation, Tokyo, Japan) by monitoring the (004) diffraction peak with respect to its position in InP.

**Figure 1:**
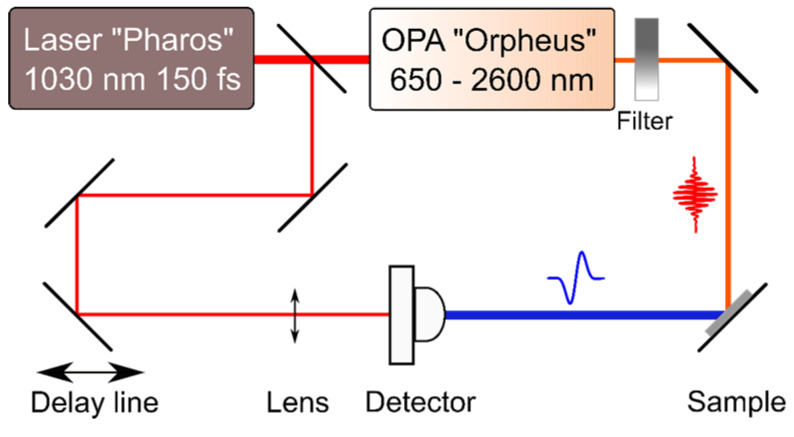
Experimental set-up of THz excitation spectra measurements.

THz-TDS experiments were performed on the samples described above. The set-up of these experiments (Figure 1) was based on an amplified Yb:KGW femtosecond laser system (PHAROS, Light Conversion Ltd., Vilnius, Lithuania) operating at 1030 nm at 200 kHz pulse repetition rate with a cavity-tuned optical parametric amplifier (OPA, ORPHEUS, Light Conversion Ltd., Vilnius, Lithuania). Optical pulses of ~160 fs duration and central wavelengths tunable from 640 nm to 2600 nm were used for sample photoexcitation, while the emitted THz pulses were detected by a GaAsBi photoconductive antenna (TeraVil Ltd., Vilnius, Lithuania) activated by a small part of the Yb:KGW laser beam.

## 3. Results

### 3.1. THz Excitation Spectra Measurements

**Figure 2:**
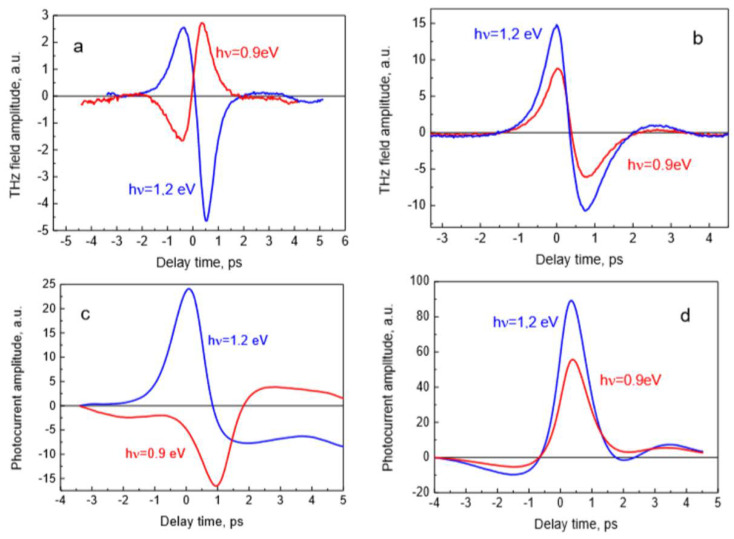
THz electric field (**a**,**b**) and photocurrent (**c**,**d**) pulses measured at two different optical wavelength beams impinging on sample A from the InP substrate side (**a**,**c**) and from the GaInAs epitaxial layer side (**b**,**d**).

Results of the measurements are illustrated in Figure 2, where the THz radiation pulses emitted from the sample A surface are shown. Femtosecond optical pulses were impinging the sample’s surface at a 45° angle from the substrate side (Figure 2a), and from the GaInAs layer-side (Figure 2b), THz pulses were measured in the quasi-reflection direction. THz pulses emitted after photoexcitation at two different optical wavelengths are presented. It can be seen that the amplitudes are almost five times higher when the optical pulses are incident on the sample from the epitaxial layer-side, which could be explained by the presence of additional reflections of both optical and THz pulses at the heterointerface in the substrate-side excitation case. Another characteristic feature of these graphs is more unexpected: the THz pulses radiated from this sample when excited from the substrate side have opposite polarities at different exciting photon energies. No such change is present when the sample is illuminated from the epitaxial layer-side.

THz pulse emission from the semiconductor surfaces is mainly caused by the ultrafast changing photocurrents leading to a dynamic electrical dipole formation [1]. These photocurrents appear either due to the built-in electric fields or due to the different electron and hole propagation velocities away from photoexcited location. As the THz field amplitude measured in the far field is proportional to the first derivative of the photocurrent in time $E_{THz\;}\sim dJ_{ph}/dt$, the temporal shape of *J_ph_* can be determined by integrating the expression. The results of integration are shown in Figure 2c,d. The photocurrent rather than the THz electric field amplitude spectra are more appropriate for the experiment and theory comparison which will be presented in this contribution later.

**Figure 3:**
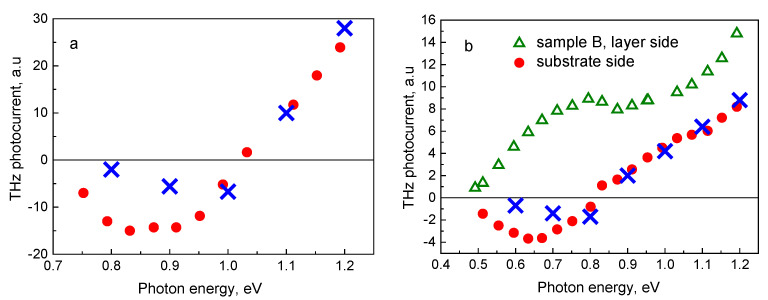
THz excitation spectra of GaInAs/InP (**a**) and GaInAsBi/InP (**b**) heterostructure samples measured for cases of the layer-side photoexcitation (empty green triangles) and the substrate-side photoexcitation (full red circles). Blue crosses show the Monte Carlo simulation results.

The experimentally determined peak photocurrent dependences on laser photon energy measured from both heterostructure samples are shown in Figure 3. The THz pulse polarity inversion is present in both samples when illuminated from the substrate side. One has to point out that in the bismide-containing layer (sample B), this effect sets on at lower photon energies than in sample A. The polarity inversion takes place at a photon energy range where a characteristic kink is observed on a THz excitation spectrum measured from the sample B epitaxial layer-side (Figure 3b—green triangles). It has been shown that similar shapes of the THz excitation spectra are indicating the onset of photoelectron propagation above the conduction band offset from the narrow-gap layer into the wide bandgap part of a heterostructure [15], which suggests that the observed polarity inversion effect is also of a similar origin. An analogous comparison of the THz excitation spectra measured from both sides of sample A is not possible, because the epitaxial layer in this sample is much thicker, and only a few of the electrons generated at the layer/air interface reach the heterointerface location.

The main part of the THz pulse is generated during the first 100–200 fs after photoexcitation, that is, at the ballistic stage of photocarriers’ movement. When analyzing THz surface emission, it is generally assumed that the transient photocurrent is directed perpendicular to the illuminated semiconductor surface, along the built-in surface electric field direction [1] or the induced spatial separation of photoexcited electrons and holes [19]. However, in cubic semiconductors, the absorption of linearly polarized radiation results in the photoexcited electron momenta from heavy-hole valence subband directed mainly in the plane perpendicular to the electric field of a light wave [11]. This optical alignment of electron momenta can also result in the appearance of a lateral (parallel to the illuminated semiconductor surface) transient photocurrent component. The THz radiation induced by this photocurrent component is predominantly directed perpendicular to the illuminated surface, and hence it will be more efficiently out-coupled from the semiconductor [12].

When the photon energy is low and electrons with excess energy below the conduction band offset *U* are excited in the narrow-gap part of the heterostructure, the photocurrent arises as a result of electron reflections from the surfaces of the epitaxial layer and is directed towards the layer/air interface. At higher photon energies, when the excess energy becomes larger than *U* and is sufficient enough to overcome the potential step, the electrons moving towards the substrate of the structure also start to take part in the photocurrent. This photocurrent has an opposite direction to the current flowing in the epitaxial layer. Starting from a certain photon energy slightly exceeding the threshold energy $\varepsilon_{g}+U$, the contribution into the photocurrent of electrons transmitted into the substrate and electrons reflected from the external surface at *z* = 0 can prevail over the photocurrent caused by the reflection of electrons from the epitaxial layer from heterointerface, which results in polarity inversion of THz field amplitude.

### 3.2. Model Calculations

Let us first consider a simple theoretical model for the formation of a photocurrent in a heterostructure excited by femtosecond laser pulses. The structure under study is a narrow-gap semiconductor layer grown on a wide-gap semiconductor substrate. We only take into account the optical transitions of electrons from the heavy-hole subband, and neglect the motion of holes. In addition, we neglect the screening effect caused by spatially redistributed photocarriers and assume that the optical pulse is instantaneous with the shape approximated by the δ-function. As the THz pulse reaches its peak during optical excitation pulse, we neglect the bulk collisions of photoelectrons.

When optical radiation is incident on the structure from the narrow-gap top layer- side located at 0≤z≤L, the kinetic equation for distribution function $f_{p}\left(z,t\right)$ of photoelectrons in this layer takes the form:

(1) $$\frac{\partial f_{p}}{\partial t}+v_{pz}\frac{\partial f_{p}}{\partial z}=W_{p}\delta \left(t\right)e^{-\alpha z},$$

where $\nu_{pz}=p_{z}/m_{1}$ is the z-component of electron velocity, *ε***_p_** is the energy of an electron with momentum **p**, *α* is the optical absorption coefficient (the photon energy is assumed to be lower than the wide-gap semiconductor’s bandgap). The rate of direct optical transitions from heavy-hole subband to conduction band $W_{p}$ is given by [11]:

(2) $$W_{p}=\frac{n}{g\left(\varepsilon_{p}\right)}\left(1-P_{2}\left(\cos\gamma \right)\right)\delta \left(\varepsilon_{p}-\varepsilon_{0}\right),$$

where *n* is the photoexcited electron density at the surface *z* = 0, $P_{2}\left(x\right)$ is the second order Legendre polynomial, *γ* is the angle between the **p**-vector and the exciting radiation’s electric field, $\varepsilon_{0}=\left(\hbar \omega -\varepsilon_{g}\right)/\left(1+m_{1}/m_{1h}\right)$ is the excess energy of photoelectrons excited by femtosecond optical pulses with photon energy of ℏω, $\varepsilon_{g}$ is the bandgap of a top-layer semiconductor, $m_{1}$ and $m_{1h}$ are the electron and heavy-hole effective masses, and $g(\varepsilon )=\sqrt{2m_{1}^{3}\varepsilon }/\left(\pi^{2}\hbar^{3}\right)$ is the density of electron states in the conduction band. We assume that for electrons at the boundary *z* = 0 that the mirror reflection condition is satisfied, and hence $f_{p_{∥},p_{z}}(z\;=\;0,t)\;=\;f_{p_{∥},-p_{z}}(z\;=\;0,t)$, $p_{∥}$, is the two-dimensional electron momentum parallel to the surface.

At the heterointerface *z* = *L*, the boundary condition is written in the form $f_{p_{∥},-p_{z}}(z\;=\;L,t)\;=\;(1\;-\;T)f_{p_{∥},p_{z}}(z\;=\;L,t)$ $p_{z}>0$, which takes into account the possibility of photoelectron penetration through the potential step into the wide-gap semiconductor. The transmission coefficient for electrons with energy ε incident on a potential step at an angle θ is written as [20]:

(3) $$T\left(\varepsilon ,\cos\theta \right)=\frac{4\sqrt{m_{1}m_{2}\varepsilon \left(\varepsilon -U\right)}\cos\theta \cos\theta_{t}}{{\left(\sqrt{m_{2}\varepsilon }\cos\theta +\sqrt{m_{1}\left(\varepsilon -U\right)}\cos\theta_{t}\right)}^{2}},$$

where $m_{2}$ is the electron effective mass in a wide-bandgap semiconductor, *U* is the conduction band offset; the angle $\theta_{t}$ between electron momentum and *z*-axis in a wide-bandgap semiconductor is determined from the relation:

(4) $$\frac{\sin\theta_{t}}{\sin\theta }=\sqrt{\frac{m_{1}\varepsilon }{m_{2}\left(\varepsilon -U\right)}}.$$

Multiple electron reflections from the boundaries of the narrow-gap layer lead to a quasiperiodic character of its motion along *z*-axis (at *T* = 0, the motion becomes strictly periodic with a period $2L/v_{pz}$). The solution to Equation (1) is found by the method of characteristics and it is represented by an infinite series of terms. Below, we neglect the multiple reflections and restrict ourselves to considering the motion of electrons at times not exceeding the time of one flight over a narrow bandgap layer ($t<L/v_{0}$ ($v_{0}={\left(2\varepsilon_{0}/m_{1}\right)}^{1/2}$). The distribution function $f_{tp}(z,t)$ of electrons transmitted into a wide-gap semiconductor (*z* > *L*) is found from the equation

(5) $$\frac{\partial f_{tp}}{\partial t}+v_{tpz}\frac{\partial f_{tp}}{\partial z}=0,v_{tpz}=p_{z}/m_{2},$$

together with the boundary condition of the electron flux continuity at *z* = *L*.

The in-plane vectors of the incident and transmitted electron momenta coincide (${p^{\prime }}_{∥}=p_{∥}$), while the *z*-component of transmitted electron momenta is determined as

(6) $${p^{\prime }}_{z}=\sqrt{2m_{2}\left(\varepsilon -U\right)}\cos\theta_{t}=\sqrt{2m_{2}\left(\varepsilon -U\right)-2m_{1}\varepsilon +p_{z}^{2}}.$$

The surface photocurrent determining the amplitude of a THz pulse is defined as

(7) $$J=-\frac{e}{4\pi^{3}\hbar^{3}m}\left(\int_{0}^{L}dz\int d^{3}ppf_{p}(z,t)+\int_{L}^{\infty }dz\int d^{3}pp^{\prime }f_{tp^{\prime }}(z,t)\right).$$

Substituting the obtained electron distribution functions into (7) and carrying out the integration over *z*, and, in a spherical coordinate system over absolute value of electron momentum *p* and azimuthal angle *φ*, we obtain the surface photocurrent components to be equal to

(8) $$\begin{array}{ll} J_{z} & =-\frac{3env_{0}^{2}t}{8}\int_{0}^{1}d\left(\cos\vartheta \right)\cos\theta \left(2-2\sin^{2}\vartheta_{r}\cos^{2}\theta -\cos^{2}\vartheta_{r}\sin^{2}\theta \right)\times [2\cos\theta \times \\ & \times \left(1-e^{-\alpha L}\right)+T(\varepsilon_{0},\cos\theta )e^{-\alpha L}\left(\cos\theta +\frac{m_{1}}{m_{2}}\sqrt{\cos^{2}\theta -\cos^{2}\theta_{cr}}\right)] \end{array},$$

(9) $$J_{x}=\frac{3env_{0}^{2}te^{-\alpha L}\sin2\vartheta_{r}}{8}\left(1-\frac{m_{1}}{m_{2}}\right)\int_{0}^{1}d\left(\cos\vartheta \right)\cos^{2}\theta \sin^{2}\theta T(\varepsilon_{0},\cos\theta )$$

where $\vartheta_{r}$ is the refraction angle of the optical radiation, which is supposed to be polarized in *xz* plane, $\theta_{cr}=\cos^{-1}\sqrt{1-m_{2}\left(\varepsilon_{0}-U\right)/\left(m_{1}\varepsilon_{0}\right)}$ is the critical angle–electrons incident at angles greater than $\theta_{cr}$ are totally reflected from the potential step. Here we assume that the optical absorption length is higher than the thickness of the top layer, and hence, in the ballistic mode which we are concerned about, the parameter $\alpha v_{0}t$ can be assumed to be small. Therefore, when introducing Equations (8) and (9), we limited ourselves to the first order in this small parameter.

Further on, for the sake of simplicity, we approximate the transmission coefficient $T(\varepsilon_{0},\cos\theta )$ by the step function: T=1 for $\theta <\theta_{cr}\;$, and T=0 for $\theta >\theta_{cr}$. After integration over the variable cosθ in (8) and (9) we get the final expressions for the surface photocurrent components

(10) $$\begin{array}{ll} J_{1z}= & -\frac{env_{0}^{2}t}{5}\{\left(1-e^{-\alpha L}\right)\left(1+\cos^{2}\theta_{r}\right)+\frac{e^{-\alpha L}}{2}[1+\cos^{2}\theta_{r}-\\ & -\frac{5s^{3}}{4}\left(2-\cos^{2}\theta_{r}\right)-\frac{3s^{5}}{4}\left(3\cos^{2}\theta_{r}-2\right)]\} \end{array}$$

(11) $$J_{2z}=-\frac{env_{0}^{2}te^{-\alpha L}}{4}\frac{m_{1}}{m_{2}}{\left(1-s^{2}\right)}^{3/2}\left(\cos^{2}\theta_{r}-\frac{\left(1-s^{2}\right)}{5}\left(3\cos^{2}\theta_{r}-2\right)\right),$$

(12) $$J_{x}=\frac{env_{0}^{2}te^{-\alpha L}\sin2\vartheta_{r}}{20}\left(1-\frac{m_{1}}{m_{2}}\right)\left(1-\frac{5}{2}s^{3}+\frac{3}{2}s^{5}\right),$$

where *s* is defined as

(13) $$s=\left\{ \begin{array}{l} 1,\varepsilon_{0}<U\\ \cos\theta_{cr},\;U\text{<}\varepsilon_{0}<U/(1-m_{1}/m_{2})\\ 0,\;\varepsilon_{0}>U/(1-m_{1}/m_{2}).\; \end{array}\right..$$

Equations (10) and (11) represent the contributions to the normal component of electron photocurrent in the upper narrow bandgap layer and in the substrate, respectively. Thus, the normal component of the total photocurrent is defined as $J_{z}=J_{1z}+J_{2z}$.

The expressions of the surface photocurrent components for the substrate-side excitation case are calculated in a similar way. The corresponding expressions for the components $J_{2z}$ and $J_{x}$ are obtained from Equations (11) and (12) by omitting the factor exp(−αL); the contribution from the top-layer photoelectrons to the normal photocurrent component is obtained from Equation (10) after removing the factor exp(−αL) before the second term in the square bracket and changing the sign in the first term.

**Figure 4:**
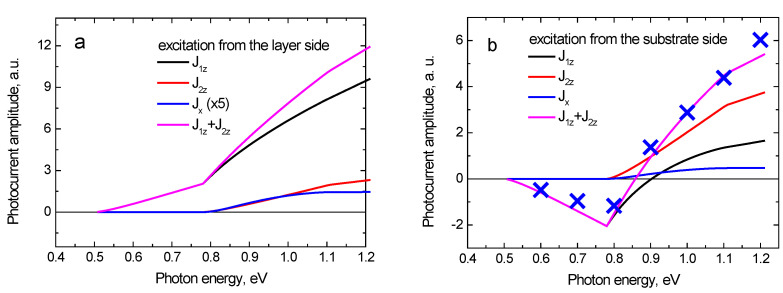
Results of the analytical calculations of different photocurrent components by use of Equations (10)–(13), for the excitation from the GaInAsBi layer side (**a**) or the InP substrate side (**b**) Blue crosses show the Monte Carlo simulation results.

Using Equations (10)–(13), we calculate the dependencies of photocurrents’ contributions on exciting photon energy (Figure 4). In the calculation, the following parameters are used: *L* = 0.24 μm, $\varepsilon_{g}$ = 0.5 eV, *U* = 0.28 eV, $m_{1}$ = 0.042 $m_{0}$ (GaInAsBi), $m_{2}$ = 0.078 $m_{2}$ (InP); the optical absorption coefficient is approximated as $\;\alpha =2.87\cdot 10^{4}\;{\left(\hbar \omega -\varepsilon_{g}\;\right)}^{\frac{1}{2}}/\hbar \omega$ [cm^−1^].

In the subthreshold photon energy range, when $\varepsilon_{0}=\hbar \omega -\varepsilon_{g}<U$, electrons cannot surmount the potential step and only the carriers in the narrow-gap layer contribute to the photocurrent. The corresponding expression follows from Equation (10) at *s* = 1 and for the excitation from the top layer-side $J_{z}=-env_{0}^{2}t\left(1-e^{-\alpha L}\right)\left(1+cos^{2}\theta_{r}\right)/5$ (for the excitation from the substrate side, this expression changes signs). It is evident that, in this case, the photocurrent tends to zero when the top layer-width is narrower than the optical penetration length (αL>1) and it disappears completely for a uniform photoelectron distribution. A slight growth of the photocurrent in the spectral region $\varepsilon_{g}<\hbar \omega <\varepsilon_{g}+U$ is caused by the dependences of parameters α and $v_{0}$ on photon energy.

When the photon energy is higher than $\varepsilon_{g}+U$, the photoelectrons acquire enough energy to pass into the wide bandgap substrate. At the excess electron energy slightly exceeding the threshold energy *U*, only a small fraction of electrons traveling almost perpendicularly to the interface pass into the substrate; most of the photoelectrons undergo reflection. With increasing excess electron energy, the angle of total internal reflection increases, therefore, the photoelectrons incident on the heterointerface at greater angles can also penetrate into the substrate. This leads to an increase in the fraction of electrons that pass into the substrate and to a steeper growth of the substrate photocurrent in the spectral range $\varepsilon_{g}+U<\hbar \omega <\varepsilon_{g}+U/\left(1-m_{1}/m_{2}\right)$. At photon energies $\hbar \omega >\varepsilon_{g}+U/\left(1-m_{1}/m_{2}\right)$ which corresponds to electron energy $\varepsilon_{0}>U/\left(1-m_{1}/m_{2}\right)$, the angle of total internal reflection is $\theta_{cr}=\pi /2$ and all electrons incident on the interface pass into the substrate.

Figure 4 shows that after exciting the heterojunction from the substrate side, the photocurrent depends on photon energy non-monotonically: at low photon energies, it is negative, and at ℏω≈0.85 eV, it changes sign to positive. This feature is explained by the fact that in the subthreshold photon energy range, photoexcited electrons in the narrow bandgap layer move away from the substrate and, therefore, their contribution to the photocurrent is negative. As the photon energy increases above the threshold value $\hbar \omega <\varepsilon_{g}+U,$ the contribution of $J_{1z}$ to the photocurrent decreases in absolute value due to a decrease in the fraction of photoelectrons reflected from the heterointerface, and changes its sign at a photon energy of ℏω≈0.9 eV. On the other hand, the contribution of photoelectrons in the substrate $J_{2z}$ is positive and rises with increasing photon energy. As a result, at photon energies lower than 0.85 eV, the total photocurrent is negative because $J_{1z}$ contribution prevails, while at higher photon energies, the dominant contribution is of those photoelectrons that are passed into the substrate, and the total photocurrent becomes positive.

As it follows from Equation (12), the anisotropy of photoelectron momentum distribution can lead to the appearance of a lateral (parallel to the heterointerface) photocurrent component $J_{x}$. This photocurrent component arises when the effective masses of electrons in the narrow-gap layer and the substrate do not coincide, which leads to a difference in the lateral components of the photoelectron velocities reflected from the interface and transmitted to the substrate. When the structure is excited from the substrate side, the photoelectrons are mainly located near the heterointerface and, in this case, the lateral photocurrent is higher than under excitation from the layer side.

Equations (10)–(13) do not describe the time dependences of photocurrents; more detailed analysis of the photoexcited carrier transport in the heterostructures was performed by the ensemble Monte Carlo (MC) method. The optical absorption coefficient, electron and hole effective masses, conduction band non-parabolicity, deformation potentials, optical phonon energies, and other parameters determining carrier scattering probabilities in GaInAsBi were assumed to be equal to those of Ga_0.47_In_0.53_As and were taken from [21]; the corresponding parameters for InP were as in [22]. The calculations for different photon energies had been carried out under the condition of a constant photon flux of $W/\hbar \omega =5\cdot 10^{11}$ cm^−2^, at ℏω=1.2 eV, it corresponds to W=0.48 μJ/cm^2^. In the calculation, an ensemble of 1,000,000 particles was used; the electric field was recalculated for each 1 fs time step, the spatial step was 1 nm. The emitted THz electric field in the far field region was defined by the time derivative of the surface transient photocurrent.

**Figure 5:**
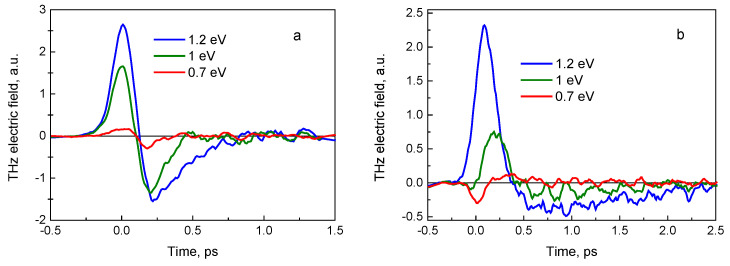
THz electric field pulses calculated by MC method for three different femtosecond optical pulse photon energies. The heterostructure is illuminated from the GaInAsBi layer-side (**a**) or from the InP substrate side (**b**).

**Figure 6:**
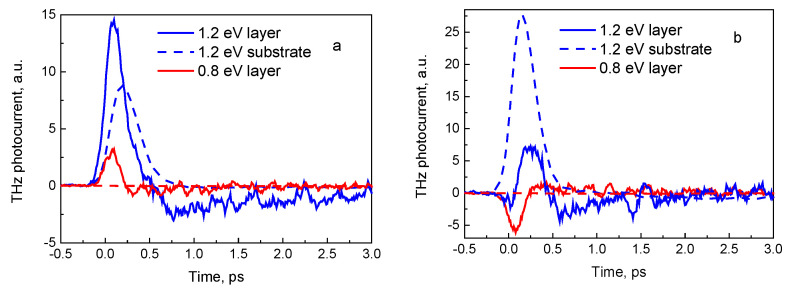
The MC calculated contributions of GaInAsBi layer (solid lines) and InP substrate (dashed lines) to the photocurrent for a heterojunction excited by femtosecond pulse optical radiation from the top layer (**a**) and from the substrate side (**b**); the blue and red lines correspond to the photon energies of 0.8 and 1.2 eV, respectively. The photocurrent in the substrate at 0.8 eV is zero.

Figure 5 shows the time dependences of THz electric field emitted from the GaInAsBi-InP heterojunction excited by 160-fs laser pulses with photon energies of 0.7, 1.0, and 1.2 eV from the layer and the InP substrate sides. It can be seen that at the excitation from the top layer-side (Figure 5a), the amplitude of a THz field monotonically grows with increasing photon energy. At photon energies higher than ~0.9 eV, this growth becomes faster because the electrons have energies sufficient to surmount the barrier at the heterostructure interface. When photoexciting from the substrate side (Figure 5b), this effect leads to the inversion of THz field polarity at higher photon energies, because of the contributions of electrons from the layer, and from the substrate to photocurrent having opposite signs. This is confirmed by the MC simulation results shown in Figure 6, where the time dependencies of photocurrents in the narrow-gap layer and the substrate are separately presented. It can be seen that in the case of excitation from bismide layer-side, both contributions to the photocurrent have the same sign (Figure 6a), while under excitation from the substrate with 0.8 eV and 1.2 eV photons, the signs of these contributions are opposite (Figure 6b). Figure 4b also shows that analytical calculations are in agreement with the MC simulation.

In Figure 6, the MC method calculated photocurrents for substrate-side excitation by femtosecond optical pulse with different photon energies are shown. It is seen that for both structures, the MC simulation shows the polarity inversion of photocurrent at photon energies close to the experimentally observed values. In the subthreshold region of exciting photon energies, the calculations lead to the lower values of photocurrent amplitude in comparison with the experimentally measured ones. This difference is most likely due to the fact that the MC simulation took into account only the electric field caused by the spatial separation of photoexcited electrons and holes, but neglected the built-in electric field near the heterojunction boundary. In the considered heterostructures, the built-in electric field would lead to an increase in the photocurrent.

## 4. Conclusions

THz radiation pulses emitted from the Ga_0.47_In_0.53_As/InP and Ga_0.47_In_0.53_As_1−x_Bi_x_/InP (x = 4%) heterostructures illuminated by femtosecond optical pulses from the narrow bandgap layer-side and from the wide bandgap substrate-side were measured for a wide range of optical pulse wavelengths. These pulses changed their polarity to the opposite when the excess energy of the photoexcited electrons became comparable with the conduction band offset in the investigated Bi-containing heterostructure. This effect was analyzed both analytically and numerically by the Monte Carlo simulation, taking into account the optical alignment of photoexcited carrier momenta and the quasi-ballistic non-equilibrium electron propagation during the THz pulse emission. Good agreement between the experiment and the theory was obtained when analyzing the results of a well-known Ga_0.47_In_0.53_As/InP heterostructure; the conduction band offset *U* = 0.28 eV was obtained for the heterostructure containing a Ga_0.47_In_0.53_As_0.96_Bi_0.04_/InP layer grown on InP substrate. These results evidence that, at least for pure A3B5 semiconductors, the carrier separation due to the optically aligned ballistic electron movement rather than their random diffusive propagation (the photo-Dember effect) is responsible for the dynamic electrical dipole formation and THz emission. Moreover, the proposed THz excitation spectroscopy can be used as a direct method to analyze the energy band line-ups in semiconductor heterostructures.

## Data Availability

Not applicable.
